# Ultrasound-Assisted Extraction of Apple Pomace: Statistical Optimization and Antioxidant Activity Evaluation

**DOI:** 10.3390/molecules31081352

**Published:** 2026-04-20

**Authors:** Magdalena Korol, Anna Łętocha, Małgorzata Tabaszewska, Łukasz Skoczylas, Elżbieta Sikora

**Affiliations:** 1 Cracow University of Technology, Faculty of Chemical Engineering and Technology, Department of Organic Chemistry and Technology, Warszawska St. 24, 31-155 Cracow, Poland; magdalena.korol@doktorant.pk.edu.pl; 2 Department of Plant Product Technology and Nutrition Hygiene, Faculty of Food Technology, University of Agriculture in Krakow, 30-149 Krakow, Poland; malgorzata.tabaszewska@urk.edu.pl (M.T.); lukasz.skoczylas@urk.edu.pl (Ł.S.); 3 Department of Human Nutrition and Metabolomics, Pomeranian Medical University in Szczecin, 71-460 Szczecin, Poland

**Keywords:** HPLC analysis, design of experiment, DPPH, Folin–Ciocalteau, antioxidant activity, extraction, polyphenols, apple pomace

## Abstract

Apple pomace, produced by the apple processing industry, is a raw material rich in bioactive compounds with a broad spectrum of biological activity, yet it remains underutilized. Both the method of raw material stabilization and the extraction technique influence the quality of the final apple pomace extract. The aim of this study was to select the most effective stabilization method and to determine optimal conditions for ultrasound-assisted extraction (UAE) of total phenolic compounds (TPC) from apple pomace. The extraction process was optimized using mathematical methods of experimental design. Input parameters included extraction temperature and time, solvent composition (ethanol concentration), and the raw material-to-solvent mass ratio. Output variables included total phenolic content and antioxidant activity of the extracts. Total phenolic content was determined using the Folin–Ciocalteu method, and antioxidant activity was determined using the DPPH (2,2-diphenyl-1-picrylhydrazyl) assay. The conditions enabling maximum extraction of phenolic compounds (total phenolic content: 8.207 mg GAE/mL and 88.09% DPPH inhibition) were achieved at 90 min, a temperature of 45 °C, an ethanol concentration of 50%, and a raw material-to-solvent ratio of 8 g.

## 1. Introduction

The need for sustainable development and environmental protection is clear in the cosmetic industry as well. Manufacturers increasingly use natural raw materials, seek sustainable production methods, and explore alternative packaging solutions to create more eco-friendly products. In this context, the management of food-processing by-products, such as pomace, fits perfectly within the “zero-waste” concept [1]. The amount of waste generated during fruit and vegetable processing ranges from 10 to 35% of the raw material mass [2], with pomace constituting the largest share [3].

**Figure 1:**
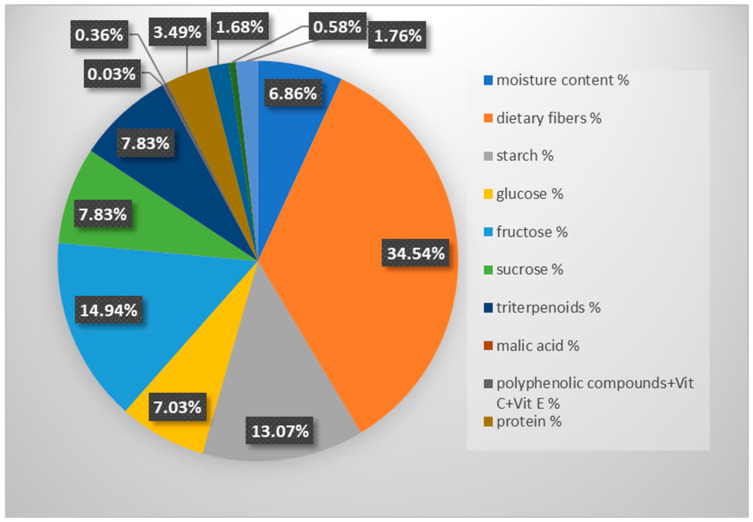
Approximate composition of dried apple pomace.

Apple pomace is the main by-product generated during apple juice production, with thousands of tons produced annually worldwide [4]. Its recycling poses a significant challenge for the fruit-processing industry. Poland, as a leading producer of organic apples, reached a production volume exceeding 4.2 million tons in 2022, representing a 5% increase compared with 2021. During the fruit-pressing process, pomace accounts for approximately 25–30% of the initial apple mass [5,6]. It consists mainly of peel and pulp (95%), seeds (2–4%), and stems (1%). Its chemical composition depends on the apple variety, origin, maturity stage, and processing parameters [7,8]. The approximate composition of dried apple pomace is shown in Figure 1 [9,10,11,12].

Extracts obtained from apple pomace are a valuable source of biologically active compounds (including carbohydrates, proteins, polyphenols, and triterpenes) with antioxidant, anti-inflammatory, and antimicrobial properties, making them an attractive natural cosmetic raw material [13,14]. Selected groups of bioactive compounds identified in apple pomace extracts are presented in Table 1 [15].

The phenolic compounds present in apple pomace exhibit strong antioxidant activity and have been shown to possess anti-inflammatory, antiproliferative, anticancer, and cardioprotective properties [16,17]. The phenolic profile of fresh apple pomace is dominated by chlorogenic acid, caffeic acid, catechin, epicatechin, rutin, and quercetin glycosides [18,19]. Dihydrochalcones such as phloretin and phlorizin show great potential as natural alternatives to synthetic antioxidants and antimicrobial agents [11]. Other constituents, including terpenoids, have also been associated with antioxidant, antibacterial, and anticancer activities [20].

The inhibitory effects of fruit pomace extracts against both Gram-positive and Gram-negative bacteria are widely described in the literature [21,22]. Apple pomace powder has demonstrated antimicrobial activity against *Micrococcus luteus* and *Bacillus subtilis* [23]. Polyphenol-rich extracts obtained from plum pomace have exhibited antimicrobial activity against *Listeria* spp., and they have shown bacteriostatic effects against *Salmonella* spp., *Listeria* spp., and *Escherichia coli* [24].

Pomace generated in the apple-processing industry, a raw material rich in bioactive compounds, is increasingly viewed as a valuable bioresource with potential applications in food, nutraceuticals, pharmaceuticals, cosmetics, and personal care formulations [25] or biodegradable packaging materials [26]. The possibility of using such industrial by-products is of great economic importance and aligns closely with the principles of the circular economy [27]. It is well established that the methods used to obtain plant raw materials strongly influence their quality. Among others, UAE is widely recognized as a novel and effective technique for recovering bioactive compounds from plant materials, including apple pomace [28,29,30]. The extraction parameters such as extraction time, temperature, and the type of solvent, significantly influence the process efficiency and the composition of the final extract [17,31]. The structure of apple pomace, rich in pectin and insoluble fiber, may limit phenolic release due to hydrogen bonding and physical entrapment. In addition, sugars and organic acids can also contribute to the Folin–Ciocalteu response. Moreover, the efficiency of UAE is influenced by matrix-related changes in pH and conductivity, as demonstrated in water–CO_2_ ultrasound extraction systems, where increasing CO_2_ concentration resulted in measurable increases in both parameters, directly affecting the extraction performance [32]. Moreover, the stabilization method (such as drying, freezing, or lyophilization) applied for plant material prior to extraction can significantly influence the final quality the extract [33,34]. Therefore, the aim of the study was to determine the influence of different stabilization methods and to optimize the UAE process of apple pomace using mathematical methods of experimental design (DoE). The novelty of this study lies in the combined evaluation of three stabilization techniques—drying, freezing, and freeze-drying—and their effect on UAE efficiency, followed by process optimization using response surface methodology. To date, studies on apple pomace have rarely investigated stabilization effects in combination with UAE optimization [35,36]. In addition, the present work focuses on apple pomace derived from the Champion cultivar, which has not previously been addressed in the context of UAE-based optimization studies. Furthermore, the optimized extract was subjected to detailed HPLC analysis, providing comprehensive insight into its phytochemical profile, an aspect not commonly included in UAE optimization studies.

Although ultrasound-assisted extraction of apple by-products has been explored previously, studies simultaneously evaluating raw material stabilization strategy, statistically optimized UAE parameters, and cultivar specific phytochemical outcomes remain limited. The novelty of the present work lies in the integrated assessment of three stabilization methods (drying, freezing, and freeze-drying) combined with DoE-based optimization of UAE and subsequent HPLC-based characterization of the optimized extract obtained from apple pomace of the Champion cultivar.

## 2. Results and Discussion

### 2.1. Study of the Influence of the Stabilization and Extraction Methods of Plant Raw Material on the Properties of Extracts

Pomace is a material characterized by low durability and stability. Due to its high water content, which can be up to 74% (Table 2), it is susceptible to microbiological contamination [37]. In addition to microbial growth, the high moisture level promotes enzymatic reactions, such as polyphenol oxidase (PPO)-mediated oxidation, which rapidly degrade phenolic compounds and deteriorate the overall quality of the material. As a result, immediate stabilization is essential to prevent spoilage, inhibit enzymatic activity, and preserve the bioactive compounds present in apple pomace [33,38].

The study examines the effect of different stabilization methods (conventional drying, freezing, and freeze-drying) on the quality of apple pomace.

Extractions of stabilized plant material were performed using two methods: continuous extraction in a Soxhlet apparatus and UAE, both using the same solvent (an aqueous ethanol solution) and a constant solid-to-solvent ratio of 1:50 (m/v).

To compare the effectiveness of both processes, the quality of the obtained extracts was tested by determining antioxidant properties using the DPPH (2,2-diphenyl-1-picrylhydrazyl) assay and the total phenolic content in the apple pomace extracts (based on the Folin-Ciocalteau method). The results shown in Table 3 represent average values obtained from three measurements.

The results indicate that freeze-drying was the most effective stabilization method, yielding the highest polyphenol content and antioxidant activity, which is in agreement with Goli et al. [38].

This is consistent with the findings of Goli et al. [38], who demonstrated that freeze-dried apple pomace exhibited the greatest antioxidant activity and the most favorable total phenolic content. Moreover, extracts obtained from material stabilized by freezing and by conventional drying show comparable antioxidant properties; however, the polyphenol content in extracts from frozen material is lower. This is likely because certain enzymes, such as polyphenol oxidase (PPO), remain active after thawing, which contributes to the degradation of phenolic compounds in the extract [38,39]. However, when the economic aspects of the process are considered, obtaining freeze-dried material is costly, whereas conventional drying remains the least expensive and technologically simplest stabilization method. Therefore, conventionally dried plant material was selected for further analyses.

### 2.2. The Influence of Input Parameters on the Total Phenolic Content (TPC) and Antioxidant Activity

The concentration of polyphenols in apple pomace is influenced by many factors, including genetic, environmental, and technological ones [20]. The extraction process is the most important step in the production of bioactive compounds from plant material and by-products [40]. In this study, continuous extraction and ultrasound-assisted extraction were compared, and ultrasonic-assisted extraction was selected as the appropriate method due to its high efficiency, energy savings, shorter process time, and suitability for isolating compounds sensitive to elevated temperatures. However, it should be emphasized that the final efficiency of the extraction process depends on many factors, in particular the type of solvent used and its concentration, which results from different affinities of individual compounds for a given extraction medium [41]. Moreover, differences in extraction techniques, methodologies applied, apple pomace processing methods, and seasonal variability may significantly influence the observed differences in the content of compounds with antioxidant properties [42,43,44,45]. Understanding and identifying the factors affecting extraction efficiency is crucial for optimizing antioxidant content in apple pomace. For this purpose, a statistical design of experiment approach was used.

It was verified whether the input parameters (extraction times, temperatures, raw material-to-solvent ratio, and solvent composition) had a significant effect on the output parameters. The total content of phenol compounds and the antioxidant activity of the extracts were classified as output parameters. Table 4 presents the specific values of process parameters and the results of the total content of phenolic compounds and the antioxidant activity of each sample, expressed as gallic acid equivalent (mg GAE/mL).

### 2.3. Results of Statistical Analysis

**Figure 2:**
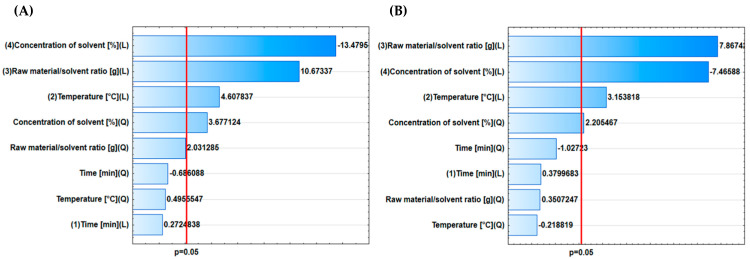
Pareto charts for the influence of input parameters on (**A**) the total phenolic content and (**B**) the antioxidant activity.

The results of the statistical analysis confirmed that the input parameters had a significant effect on the output parameters in the case of apple pomace extracts. Figure 2 presents the Pareto charts for the applied independent variables. The red line indicates the threshold for statistically significant results at *p* < 0.05. The results showed that the parameters exerting a statistically significant impact on the total phenolic content and antioxidant activity were ethanol concentration (solvent composition) (in both linear and quadratic terms), the raw material-to-solvent ratio (linear term), and the temperature (linear term). These results are consistent with those of other research groups that have optimized extraction from olive [46] and loquat leaves [47]. The extraction time was not statistically significant, likely because the investigated range had been optimized in preliminary experiments and already fell within the optimal operational window. As a result, further changes to this parameter did not lead to statistically significant differences in the extraction efficiency or outcomes.

**Figure 3:**
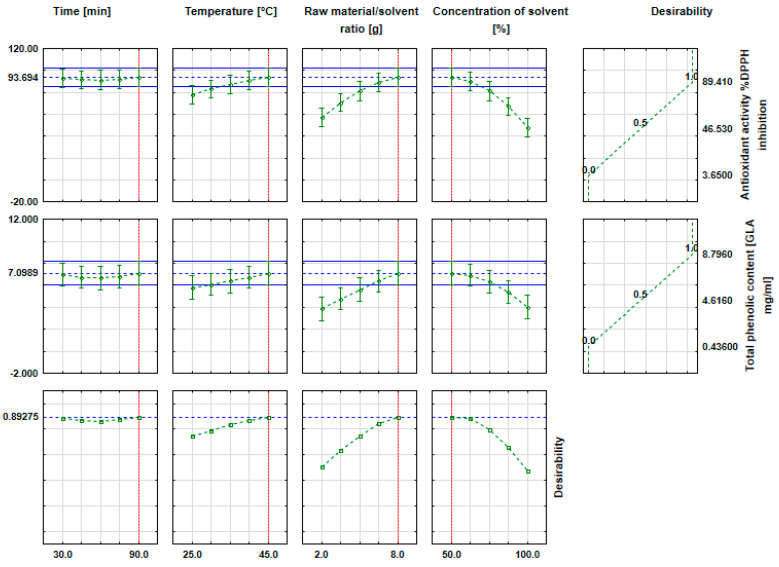
Approximation profiles for the influence of input parameters on: total phenolic content and antioxidant activity.

The next step of the statistical analysis was the preparation of approximation profiles (Figure 3), indicating which values of the independent parameters allow for the most desirable values of the dependent variables to be achieved. The aim of the study was to develop the apple pomace extract characterized by the highest phenolic content and the greatest antioxidant activity.

The analysis of the approximation profiles (Figure 3) shows that the apple pomace extract with the highest total phenolic content (approx. 8.796 mg GAE/mL) and the greatest antioxidant activity (approx. 89.410% DPPH inhibition) was obtained under the following conditions: the temperature of 45 °C, the extraction time of 90 min, the concentration of ethanol of (50%), and the raw material-to-solvent ratio of 8 g. The superior extraction performance at 50% ethanol can be attributed to optimal solvent polarity, enabling efficient solubilization of both phenolic acids and flavonoids [48]. Moderate temperature (45 °C) enhances diffusion and cavitation while limiting thermal degradation, as reported also for UAE kinetics [49].

The analysis of the approximation profiles shows similar relationships in terms of the influence on both the antioxidant activity of the samples and their total polyphenol content, which is consistent with findings reported by other research groups [50,51]. Egüés et al. [52] reported that, at high polyphenol concentrations, antioxidant capacity measured by DPPH may decrease due to thermal degradation effects. This outcome may be attributed to the fact that the quality of the extracted phenolic compounds and flavonoids can deteriorate at elevated temperatures, with the greatest percentage reduction observed in samples extracted at lower temperature (40 °C). Consequently, samples processed at a higher temperature range (65 and 90 °C) may exhibit lower antioxidant capacity when assessed using the DPPH assay. Elevated temperatures also reduce cavitation efficiency by increasing vapor pressure inside bubbles, further limiting UAE effectiveness [49]. It should be emphasized that the present interpretation focuses on process-related and physicochemical factors governing UAE efficiency, such as solvent polarity, diffusion, and cavitation behavior. While a detailed molecular-level analysis of individual radical-scavenging pathways would require complementary techniques, the discussion provided here offers a robust mechanistic rationale appropriate for process optimization studies.

The graphs also show that the total phenolic content and antioxidant activity decreased with increasing the concentration of solvent. This is consistent with studies by other research groups, where the use of ethanol (60%) gave better results than the use of 96% ethanol [50]. Comparative studies have shown that although methanol often provides higher antioxidant yields, aqueous and ethanol-based systems can achieve comparable DPPH activity under optimized conditions [52,53]. Given toxicity and environmental concerns, ethanol remains a preferred green solvent [54]. From an economic perspective and ecological considerations, the use of distilled water instead of ethanol is also advantageous. However, distilled water was not the least efficient solvent, as the lowest extraction efficiency in all three analyses was observed with 96% ethanol. The analysis of the graph (Figure 3) also shows that using lower temperatures and smaller amounts of raw material adversely affected the values of the analyzed parameters. Conversely, the extraction time had no significant effect on the total polyphenol content or antioxidant activity. Different relationships were reported by other authors, where the ABTS (2,2′-Azino-bis (3-ethylbenzothiazoline-6-sulfonic acid) assay showed the highest activity at the shortest extraction time, while the highest of total phenolic content (TPC) and DPPH values were obtained at an intermediate time [29]. The decrease in ABTS activity with extended extraction times may be related to the degradation of certain antioxidant compounds [55,56]. In turn, high ABTS values at short extraction times may result from the rapid reaction of some antioxidants, while others require longer extraction time to reach optimal activity [57]. It should be emphasized that the DPPH and ABTS assays may differ in their selectivity toward specific antioxidants and in reaction kinetics, which are influenced by the chemical properties of the compounds, interactions with the matrix, and extraction conditions such as temperature, time, and ultrasound intensity [57,58,59,60].

Based on the obtained results and the design of experiment (DOE), extraction parameters were selected to produce high-yield apple pomace extracts with strong antioxidant properties. As a result, extract samples were prepared (Table 5), and their characteristics were evaluated in subsequent analyses.

The use of optimized parameters resulted in extracts with antioxidant activity of 88.09% and a total polyphenolic content of 8.2 mg/mL. The obtained values are comparable to the literature data, although significant differences in the optimal extraction conditions can be observed. For instance, Frontuto et al. [61] optimized ultrasonic extraction from apple pomace using 70% ethanol and identified an amplitude of 82.36%, extraction time of 35.24 min, and temperature of 51.48 °C as optimal conditions, yielding 74.53 mg GAE/100 g of raw material. Egües et al. [52], using water as a solvent, determined that 20 min, 90 °C, and 50% amplitude ensured the maximum content of phenolic compounds (6.07 mg GAE/g). In comparison with these studies, it is noteworthy that in the present work, high content of polyphenols and antioxidant activity were achieved at a moderate extraction temperature, which is particularly important due to the thermolability of many phenolic compounds, including anthocyanins and flavonols such as quercetin, rutin, and catechin [62,63]. The use of milder temperature conditions likely reduced the degradation of these compounds, contributing to the high antioxidant activity of the extracts obtained in this study.

### 2.4. Analysis of the Phytochemical Composition of Apple Pomace Extract (HPLC)

Based on the optimized process parameters, apple pomace extract was obtained and subsequently analyzed using HPLC. Compounds were identified based on their retention times and spectra, and their concentrations were calculated using calibration curves of the respective standards. The identified compounds, presented in Table 6, belong to the class of polyphenols, a group of bioactive constituents widely recognized for their antioxidant activity.

The chromatographic results are consistent with previously published data [36], confirming that polyphenols are the predominant group of compounds in apple pomace. Among them, phloridzin is identified as one of the dominant phenolics and, according to the literature, represents 70–90% of the total phloridzin content in most apple varieties [64,65,66]. Additionally, rutin is the most abundant flavonoid in the extract, and it is well known for its strong antioxidant properties.

Compared with the available literature, the present extract contained a moderate fraction of phenolic acids, such as chlorogenic acid, and a comparatively lower contribution of flavonols (e.g., quercetin derivatives). RP-HPLC-DAD (reverse-phase high-performance liquid chromatography with diode-array detection) analyses of industrial apple pomaces from various cultivars commonly report quercetin, phloretin, and phloridzin as the dominant phenolics, with phloridzin concentrations typically ranging from 0.62 to 2.0 µg/mg dry weight [12]. Similarly, HPLC-MS studies on pomace from cultivars such as Golden Delicious identify flavonols, phenolic acids, and procyanidins as major components, although their relative abundance varies markedly depending on extraction technique and solvent system [67].

The slightly reduced abundance of certain phenolic acids and flavonols in the present extract may reflect the optimization strategy adopted in this study, which prioritized the overall antioxidant activity rather than maximal recovery of individual phenolics. Comparable trends have been reported in the UAE-optimized systems, where moderate temperatures and shorter extraction times favor the recovery of more labile dihydrochalcones while limiting diffusion-controlled extraction of less soluble flavonols. In contrast, microwave-assisted extraction often yields higher concentrations of procyanidin B2 and phenolic acids, albeit at the expense of higher thermal input [68].

A critical review of the available literature further reveals that most studies on the UAE of apple pomace focus on unspecified industrial mixtures or on cultivars other than Champion, while cultivar identity is often omitted or treated as a secondary variable. To date, data on the Champion cultivar are scarce and largely limited to studies on fresh apples, where this cultivar has been reported to exhibit high flavonol content and comparatively low levels of phenolic acids [69]. More recently, apple pomace derived from the Champion cultivar has been investigated using alternative extraction approaches, such as micellar extraction, which confirmed its favorable phenolic profile; however, these studies did not address ultrasound-assisted extraction, or the influence of UAE parameters on phenolic recovery [70]. Consequently, systematic data describing the phenolic composition of Champion apple pomace obtained under the UAE conditions remain unavailable.

Given that phenolic composition in apple pomace is strongly influenced by cultivar-dependent tissue distribution—particularly the enrichment of peel-derived compounds—the lack of Champion-specific pomace data represents a clear gap in the literature. Taken together, the present results provide, to our knowledge, the first detailed HPLC-based characterization of phenolics extracted from Champion apple pomace under optimized UAE conditions. By addressing a cultivar-specific knowledge gap and combining controlled stabilization with statistically optimized extraction, this study offers a valuable reference for future comparisons of extraction technologies and cultivar effects in apple pomace valorization.

It should be noted that the present discussion is intentionally focused on process-related and physicochemical aspects of UAE efficiency. While detailed molecular-level mechanisms (e.g., radical-scavenging pathways or cell-wall disruption kinetics) would require complementary analytical approaches, the current results provide a robust and practically relevant framework for process optimization.

## 3. Materials and Methods

### 3.1. Materials

Fresh apple pomace of the Champion cultivar was obtained in September 2022 from a local fruit-processing company located in Ryki, Poland (Kampol-Fruit Sp. z o.o.). All chemicals used in this study were of analytical grade or of HPLC-grade purity. Ethanol (99.8%, analytical grade), Folin–Ciocalteu reagent, the DPPH radical, and gallic acid were obtained from Avantor Performance Materials Poland (Gliwice, Poland) and Sigma-Aldrich (Poznań, Poland). The phenolic standards used for HPLC identification and quantification—rutin, phloridzin, quercetin, ellagic acid, 4-hydroxybenzoic acid, chlorogenic acid, 3-hydroxybenzoic acid, (+)-catechin, protocatechuic acid, ferulic acid, p-coumaric acid, gallic acid, and caffeic acid—were purchased from Sigma-Aldrich and were of ≥98% purity (HPLC-grade). Deionized water was used as a solvent (Millipore Direct-Q 3 UV, Merck, Darmstadt, Germany).

### 3.2. Methods of Stabilizing Plant Raw Materials

To assess the effect of the stabilization method on the quality of the extract, three different methods were used: freeze-drying (Martin Christ Alpha 1–2 LD plus freeze-dryer, Osterode am Harz, Germany), conventional drying (IKA Oven 125 Basic laboratory dryer, Warsaw, Poland), and freezing (Samsung freezer, Suwon, Republic of Korea). The stabilization parameters for the plant material are presented in Table 7. The dried plant material was ground and stored in an airtight, black bottle at room temperature until the analysis. The frozen and freeze-dried material was stored in airtight containers in a freezer (T = −20 °C) until the analysis began.

### 3.3. Moisture Testing of Plant Material

The pre-stabilized plant material was subjected to water content determination. The moisture content of the plant material was analyzed using an OHAUS MB25 moisture analyzer (Nänikon, Switzerland). Moisture content was determined by mass loss over time at 50 °C, with drying continued until constant mass was achieved. The final water content determination result was the average value from three replicates.

### 3.4. Extraction Methods Used in the Research

In order to select the most suitable method for subsequent optimization aimed at obtaining apple pomace extract with the best properties, a preliminary comparison of two extraction techniques was carried out: Soxhlet extraction and ultrasound-assisted process (UAE).

#### 3.4.1. Soxhlet Extraction

Before the extraction, each batch of apple pomace was ground using a mortar and pestle to obtain a particle size of less than 1 mm, which was confirmed by sieving the material through a stainless-steel sieve with a 1 mm mesh size. The powdered plant material (apple pomace: dried, freeze-dried, frozen) was weighed in an amount of 5 g and placed into to a filter paper thimble. The entire mixture was transferred to a Soxhlet apparatus and subjected to a 4 h extraction at 25 °C. As the extraction solvent, 250 mL of analytically pure ethanol with a concentration of 99.8% was used. The resulting ethanol extract was filtered using a vacuum pump, and then the solvent was evaporated in a Buchi R-80 vacuum evaporator (Buchi, Flawil, Switzerland) at a temperature not exceeding 50 °C.

#### 3.4.2. Ultrasound-Assisted Extraction

Before the extraction, each batch of apple pomace was ground using a mortar and pestle to obtain a particle size of less than 1 mm, which was confirmed by sieving the material through a stainless-steel sieve with a 1 mm mesh size. The prepared plant material (apple pomace: dried, freeze-dried, frozen) was weighed in an amount of 5 g and then transferred to a round-bottom flask and placed in an Intersonic IS-3 ultrasonic bath (Intersonic s.c., Olsztyn, Poland). The process was carried out at a frequency of 50 Hz and a power of 300 W. The extractions were carried out for 4 h at 25 °C. The 99.8% ethanol, in an amount of 250 mL, was also used as the solvent in the UAE process, maintaining a constant mass ratio of the plant material to the eluent. The resulting ethanol extract was evaporated in a vacuum evaporator at a temperature not exceeding 50 °C.

### 3.5. Study of the Apple Pomace Properties

#### 3.5.1. Measurement of Polyphenol Contents

The measurement of polyphenol content (PC) was carried out using a colorimetric Folin–Ciocalteu method at 765 nm. A total of 1 mL of liquid extract was mixed with 5 mL of Folin–Ciocalteu reagent (diluted 10-fold in water); after 4 min, 4 mL of 7.5% (m/m) Na_2_CO_3_ solution was added. The above mixture was incubated at 25 °C in the darkness for 60 min. The mixtures’ absorbance values were measured at 765 nm. A standard curve was created using gallic acid.

#### 3.5.2. Antioxidant Activity Assays (DPPH Method)

A total of 0.2 mL of liquid extract was mixed with 3 mL of 0.005% ethanol solution containing DPPH and stored for 30 min in the dark environment at room temperature. The antioxidant capacity was determined at 517 nm using a UV-vis spectrophotometer (Nanocolor UV/VIS II, Macherey-Nagel Gmbh & Co., KG, Düren, Germany).

### 3.6. Optimization Process

Based on the preliminary comparison of the extraction methods and the results obtained, ultrasound-assisted extraction was selected for optimization. The process of extraction was optimized using the mathematical methods of experiment planning. All statistical analyses and graphical outputs (Pareto charts and desirability profiles) were generated using Statistica^®^ ver. 13 (StatSoft, Cracow, Poland). The aim of the optimization was to determine the most optimal conditions for the UAE of total phenolic compounds and antioxidant activity of apple pomace. To obtain extracts using the statistical method of experimental design (DOE), the central-composition design 3^(K-p)^ was used, where p always takes the value 1 and K is the number of variables. In all cases, the statistical significance level was assumed to be *p* < 0.05. Through these profiles, it was possible to determine the changes in the dependent parameters when changing the values of the independent parameters. The group of input (independent variables) parameters included: temperature, and extraction time, solvent with varying ethanol concentration, the ratio of mass raw material to solvent, whereas the output (dependent variables) was the total content of phenol compounds, and the antioxidant activity of the extracts. The total phenolic content was determined according to the Folin–Ciocalteau method, and the antioxidant activity was determined using the DPPH method. The ranges of the variability of the process input variables are listed in Table 8.

### 3.7. Determination of the Phytochemical Composition of Apple Pomace Extract Using the HPLC Method

The analysis of the phytochemical composition of apple pomace extract obtained under optimized extraction conditions was performed based on the method described by Klimczak [71], with modifications by Tabaszewska and Najgebauer-Lejko [72]. The ground samples were placed into Eppendorf-type centrifuge tubes, followed by the addition of distilled water and mixing using a vortex mixer (Labnet, Edison, NJ, USA) for 30 s. After mixing, the samples were subjected to sonication for 15 min at 20 °C, at 40 kHz ± 2, Type IS-14 (Intersonic s.c., Olsztyn, Poland). HPLC-grade methanol containing 1% (*v*/*w*) ascorbic acid was then added to the prepared samples. The samples were mixed again using a vortex mixer, followed by sonication for 15 min at 20 °C, at 40 kHz ± 2, and then they were centrifuged for 20 min at 18,000 rpm at 4 °C (MPW-260R centrifuge, Warsaw, Poland). The supernatant was filtered using PTFE-L syringe filters with a pore size of 0.22 µm. The chromatographic analysis was performed using a Dionex UltiMate 3000 HPLC system (Waltham, MA, USA) with a Thermo Scientific DAD detector (Germering, Germany). A Cosmosil 5C18-MS-II 250 × 4.6 mm ID, 5 µm column (Nacalai Tesque, Inc., Kyoto, Japan) was used. The analysis time was 50 min, with a flow rate of 1 mL/min. The mobile phase consisted of: (A) an aqueous solution of acetic acid at a concentration of 2%, and (B) methanol at 100%. The eluent gradient was as follows: 70% eluent for 10 min; 50% at 25 min; 30% at 35 min; 95% at 40 min; and at least 95% until the end of the analysis. The detection was performed at the following wavelengths: λ = 245 nm, λ = 280 nm, λ = 320 nm, and λ = 360 nm. The determination was carried out in four replicates. The compounds were identified based on retention time and spectra, and their content was calculated using calibration curves of the respective standards.

### 3.8. Statistical Analysis

All data regarding moisture content in apple pomace stabilized by different methods, antioxidant activity, total polyphenol content, and compounds identified in apple pomace extract were presented as means from different experiments ± SD. The differences between the calculated means for each group were determined using one-way ANOVA tests using the Statistica ver. 13 statistical software from StatSoft, Kraków. All statistically significant effects discussed in the manuscript were confirmed at *p* < 0.05, and model adequacy was verified based on the consistency between experimental and predicted values.

## 4. Conclusions

To summarize, apple pomace represents one of the most abundant by-products generated during apple juice production, with thousands of tons produced annually worldwide. Its disposal remains a considerable challenge for the food industry. Due to its high content of bioactive compounds, including polyphenols, flavonoids, and carbohydrates, apple pomace offers significant potential as a valuable raw material within circular economy and sustainable processing frameworks.

By combining stabilization strategy selection with statistically optimized UAE and detailed phytochemical profiling, this study goes beyond isolated optimization approaches commonly reported in the literature. In particular, the cultivar-specific focus on Champion apple pomace provides reference data that can facilitate future comparisons of extraction technologies and raw material variability.

The findings of this study demonstrate that both the stabilization method of the raw material and the extraction parameters substantially influence the quality and bioactivity of the extracts. The obtained results show that the recommended optimal conditions for the extraction of apple pomace were the temperature of 45 °C, extraction time of 90 min, the use of 50% ethanol as the eluent, and a raw material-to-solvent ratio of 8 g/250 mL. These conditions enabled the production of extracts with high total phenolic content and strong antioxidant activity. Among all investigated variables, the solvent concentration and the raw material-to-solvent ratio were identified as statistically significant factors affecting extraction efficiency. Compared with conventional and emerging extraction methods, the UAE process used in our study offers a favorable balance between scalability and economic feasibility, the extract properties confirming its suitability as a potential functional ingredient. These results highlight the feasibility of transforming apple pomace, a low-value by-product, into a high-value source of bioactive substances for cosmetic applications.

The scope of the present work was intentionally focused on upstream processing and extract quality; therefore, biological activity at the cellular or formulation level was beyond its aim and constitutes a logical direction for future studies.

Future work should explore the stability of the extract in cosmetic formulations, its bioavailability, dermatological safety, and potential synergistic effects with other natural antioxidants. Such studies could further support the industrial implementation of the apple pomace extracts and contribute to the development of environmentally friendly, high-performance cosmetic products.

## Figures and Tables

**Table 1:** Content of some bioactive compounds present in apple pomace.

Class	Concentration (mg/kg Dry Weight)	Compounds
Phenolic acids	523–1542	Chlorogenic acid, caffeic acid, ferulic acid, p-coumaric acid, sinapic acid, p-coumaroyl-quinic acid
Flavonoids	2153–3734	isorhamnetin, kaempferol, guercetin, rhamnetin, procyanidin B2, epicatechin
Anthocyanins	50–130	cyanidin-3-O-galactoside
Dihydrohalcones	688–2535	phlorizin, phloretein

**Table 2:** Moisture content of apple pomace stabilized by various methods (*n* = 3, ±SD).

Raw Material–Stabilization Method	Freeze-Drying	Drying	Freezing
Moisture [%]	3.95 ± 0.05	0.46 ± 0.03	74.60 ± 0.26

**Table 3:** Results of the antioxidant activity and total polyphenol content in apple pomace extracts (*n* = 3, ±SD).

Extraction Type	Soxhlet Extraction			Ultrasound-Assisted Extraction		
Raw Material–Stabilization Method	Freeze-Drying	Drying	Freezing	Freeze-Drying	Drying	Freezing
Antioxidant activity [% DPPH inhibition]	75.03 ± 0.24	41.52 ± 0.17	39.11 ± 0.22	69.87 ± 0.11	40.92 ± 0.24	52.58 ± 0.27
Total polyphenol content [mg GAE/mL]	4.67 ± 0.05	4.07 ± 0.03	2.40 ± 0.01	6.61 ± 0.01	6.11 ± 0.01	2.36 ± 0.01

**Table 4:** Matrix of the experimental design and experimental data obtained for the dependent variables.

No. of the System	Input Parameters				Output Parameters	
	Time [min]	Temperature [°C]	Raw Material-to-Solvent Ratio * [g]	Solvent Concentration [%]	Antioxidant Activity [%DPPH Inhibition]	Total Phenolic Content [mg GAE/mL]
3	30	25	8	75	71.09 ± 0.08	5.207 ± 0.011
10	60	25	2	100 **	3.65 ± 0.20	0.436 ± 0.009
19	90	25	2	75	22.74 ± 0.03	1.539 ± 0.001
24	90	35	8	75	78.57 ± 0.14	5.666 ± 0.002
28	60	35	5	75	69.39 ± 0.06	4.297 ± 0.003
8	30	45	5	50	86.51 ± 0.31	5.351 ± 0.008
11	60	25	5	75	47.91 ± 0.09	2.840 ± 0.002
27	90	45	8	50	89.41 ± 0.14	8.796 ± 0.014
21	90	25	8	100	26.26 ± 0.08	1.895 ± 0.001
26	90	45	5	75	78.19 ± 0.11	5.363 ± 0.002
16	60	45	2	50	57.09 ± 0.08	2.924 ± 0.001
18	60	45	8	75	81.73 ± 0.19	6.129 ± 0.003
7	30	45	2	75	30.08 ± 0.22	2.447 ± 0.008
2	30	25	5	100	20.26 ± 0.26	1.607 ± 0.003
20	90	25	5	50	60.21 ± 0.14	3.772 ± 0.004
5	30	35	5	75	59.01 ± 0.03	3.455 ± 0.004
9	30	45	8	100	43.80 ± 0.11	2.231 ± 0.008
14	60	35	5	50	65.76 ± 0.14	4.258 ± 0.014
13	60	35	2	75	29.11 ± 0.08	1.584 ± 0.009
23	90	35	5	100	25.92 ± 0.06	1.744 ± 0.001
25	90	45	2	100	17.92 ± 0.14	1.125 ± 0.012
17	60	45	5	100	26.26 ± 0.12	1.981 ± 0.002
6	30	35	8	50	88.52 ± 0.05	7.218 ± 0.009
12	60	25	8	50	78.21 ± 0.06	5.221 ± 0.003
1	30	25	2	50	38.95 ± 0.14	2.135 ± 0.005
4	30	35	2	100	7.92 ± 0.27	0.985 ± 0.003
15	60	35	8	100	33.42 ± 0.17	2.131 ± 0.012
22	90	35	2	50	55.30 ± 0.22	2.145 ± 0.002

* all extractions were performed using 250 mL of solvent. ** anhydrous ethanol (99.8%) was used for the reaction.

**Table 5:** Extract input and output parameter values were optimized.

Input Parameters				Output Parameters	
Time [min]	Temperature [°C]	Raw Material-to-Solvent Ratio [g]	Time [min]	Antioxidant Activity [%DPPH Inhibition]	Total Phenolic Content [mg GAE/mL]
90	45	8	50	88.09 ± 0.08	8.207 ± 0.011

**Table 6:** Summary of compounds identified in the apple pomace extract obtained through the extraction process carried out under optimized conditions (*n* = 4, ±SD).

Name of Compound	Content [mg/100 g]
Rutin	1719.3 ± 16.4
Phloridzin	844.1 ± 21.9
Quercetin	427.8 ± 17.2
Ellagic acid	280.6 ± 5.6
4-Hydroxybenzoic acid	276.5 ± 3.7
Chlorogenic acid	262.0 ± 1.1
3-Hydroxybenzoic acid	188.5 ± 2.9
(+)-catechin	179.1 ± 2.7
Protocatechuic acid	94.7 ± 1.2
Ferulic acid	72.5 ± 1.6
p-coumaric acid	28.3 ± 0.3
Gallic acid	20.0 ± 0.3
Caffeic acid	18.7 ± 0.3

**Table 7:** Parameters of apple pomace stabilization processes used to obtain extracts.

Method of Stabilizing Plant Material	Temperature [°C]	Time [h]
Freeze-drying	−40 °C	24
Drying	50 °C	40
Freezing	−20 °C	24

**Table 8:** The variability ranges of independent parameters.

Independent Variable	The Ranges of Variability
Temperature [°C]	25, 35, 45
Extraction time [min]	30, 60, 90
Concentration of solvent [%]	50, 75 100
Raw material-to-solvent ratio [g]	2, 5, 8

## Data Availability

Data are contained within the article.

## References

[1] Franco L., Lopes S.T., Nazari M.T., Biduski B., Pinto V.Z., Sena J., Elita T., Ruschel L. (2023). Fruit Pomace as a Promising Source to Obtain Biocompounds with Antibacterial Activity. Crit. Rev. Food Sci. Nutr..

[2] Dilucia F., Lacivita V., Conte A., Del Nobile M.A. (2020). Sustainable Use of Fruit and Vegetable By-Products to Enhance Food Packaging Performance. Foods.

[3] Cybulska J., Zdunek A., Sitkiewicz S., Galus S., Janiszewska E., Łaba S., Nowacka M. (2013). The Possibilities of Recycling of Pomace and Other Wastes from Fruit and Vegetable Industry. Przem. Ferment. Owocowo-Warzywny.

[4] Łusiak P., Mazur J., Sobczak P., Zawiślak K., Panasiewicz M. (2023). The Use of Carrot and Apple Pomace in the Production of Healthy Snack Bars. Agric. Eng..

[5] WAPA The World Apple and Pear Association. http://www.wapa-association.org/asp/index.asp.

[6] Statistics Poland. https://stat.gov.pl/en/topics/agriculture-forestry/agriculture/rural-areas-in-poland-2018,3,4.html.

[7] Skinner R.C., Warren D.C., Naveed M., Agarwal G., Benedito V.A., Tou J.C. (2019). Apple Pomace Improves Liver and Adipose Inflammatory and Antioxidant Status in Young Female Rats Consuming a Western Diet. J. Funct. Foods.

[8] Waldbauer K., McKinnon R., Kopp B. (2017). Apple Pomace as Potential Source of Natural Active Compounds. Planta Med..

[9] Gazalli H., Malik A.H., Jalal H., Afshan S., Mir A. (2013). Proximate Composition of Carrot Powder and Apple Pomace Powder. Int. J. Food Nutr. Saf..

[10] Sato M.F., Vieira R.G., Zardo D.M., Falcão L.D., Nogueira A., Wosiacki G. (2010). Apple Pomace from Eleven Cultivars: An Approach to Identify Sources of Bioactive Compounds. Acta Sci.-Agron..

[11] Queji M.D., Wosiacki G., Cordeiro G.A., Peralta-Zamora P.G., Nagata N. (2010). Determination of Simple Sugars, Malic Acid and Total Phenolic Compounds in Apple Pomace by Infrared Spectroscopy and PLSR. Int. J. Food Sci. Technol..

[12] Rana S., Rana A., Gulati A., Bhushan S. (2014). RP-HPLC-DAD Determination of Phenolics in Industrial Apple Pomace. Food Anal. Methods.

[13] Korol M., Sikora E. (2023). Apple Pomace—Alternative Applications of Plant Extracts. Przem. Chem..

[14] Barreira J.C.M., Arraibi A.A., Ferreira I.C.F.R. (2019). Bioactive and Functional Compounds in Apple Pomace from Juice and Cider Manufacturing: Potential Use in Dermal Formulations. Trends Food Sci. Technol..

[15] Lyu F., Luiz S.F., Rosane D., Azeredo P., Cruz A.G., Ajlouni S., Ranadheera C.S. (2020). Apple Pomace as a Functional and Healthy Ingredient in Food Products: A Review. Processes.

[16] Wulandari L., Retnaningtyas Y., Lukman H. (2016). Analysis of Flavonoid in Medicinal Plant Extract Using Infrared Spectroscopy and Chemometrics. J. Anal. Methods Chem..

[17] Naczk M., Shahidi F. (2006). Phenolics in Cereals, Fruits and Vegetables: Occurrence, Extraction and Analysis. J. Pharm. Biomed. Anal..

[18] Ćetković G., Čanadanović-Brunet J., Djilas S., Savatović S., Mandić A., Tumbas V. (2008). Assessment of Polyphenolic Content and in vitro Antiradical Characteristics of Apple Pomace. Food Chem..

[19] Górnaś P., Mišina I., Olšteine A., Krasnova I., Pugajeva I., Lacis G., Siger A., Michalak M., Soliven A., Segliņa D. (2015). Phenolic Compounds in Different Fruit Parts of Crab Apple: Dihydrochalcones as Promising Quality Markers of Industrial Apple Pomace by-Products. Ind. Crops Prod..

[20] Antonic B., Jancikova S., Dordevic D. (2020). Apple Pomace as Food Fortification Ingredient: A Systematic Review and Meta-Analysis. J. Food Sci..

[21] Radenkovs V., Kviesis J., Juhnevica-Radenkova K., Valdovska A., Püssa T., Klavins M., Drudze I. (2018). Valorization of Wild Apple (*Malus* spp.) by-Products as a Source of Essential Fatty Acids, Tocopherols and Phytosterols with Antimicrobial Activity. Plants.

[22] Velićanski A.S., Cvetković D.D., Markov S.L. (2012). Screening of Antibacterial Activity of Raspberry (*Rubus idaeus* L.) Fruit and Pomace Extracts. Acta Period. Technol..

[23] Kuznetsova E., Emelyanov A., Klimova E., Bychkova T., Vinokurov A., Selifonova N., Zomitev V., Brindza J. (2017). Antioxidant, Antimicrobial Activity and Mineral Composition of Low-Temperature Fractioning Products of *Malus domestica* Borkh (Common Antonovka). Potravin. Slovak J. Food Sci..

[24] Sójka M., Kołodziejczyk K., Milala J., Abadias M., Viñas I., Guyot S., Baron A. (2015). Composition and Properties of the Polyphenolic Extracts Obtained from Industrial Plum Pomaces. J. Funct. Foods.

[25] Vandorou M., Plakidis C., Tsompanidou I.M., Adamantidi T., Panagopoulou E.A., Tsoupras A. (2024). A Review on Apple Pomace Bioactives for Natural Functional Food and Cosmetic Products with Therapeutic Health-Promoting Properties. Int. J. Mol. Sci..

[26] Asma U., Morozova K., Ferrentino G., Scampicchio M. (2023). Apples and Apple By-Products: Antioxidant Properties and Food Applications. Antioxidants.

[27] Foundation Ellen MacArthur (2015). Towards a Circular Economy: Business Rationale for an Accelerated Transition.

[28] Rubashvili I., Tsitsagi M., Chkhaidze M., Ebralidze K. (2024). Ultrasound-Assisted Extraction- and Liquid Chromatography-Based Method Development and Validation for Obtaining and Qualitative Determination of Apple Pomace Three Triterpene Acids Using Analytical Quality by Design. J. Integr. OMICS.

[29] Malenica D., Maciel L.S., Herodes K., Kass M., Bhat R. (2024). Optimization of Ultrasonic-Assisted Extraction of Antioxidants in Apple Pomace (Var. *Belorusskoje malinovoje*) Using Response Surface Methodology: Scope and Opportunity to Develop as a Potential Feed Supplement or Feed Ingredient. Sustainability.

[30] Fraterrigo Garofalo S., Demichelis F., Peletti V., Picco L., Tommasi T., Fino D. (2025). Comparative Study of Polyphenol Extraction Using Physical Techniques and Water as a Solvent: A Sustainable Approach for the Valorization of Apple Pomace. Environ. Sci. Pollut. Res..

[31] Zhang Q.W., Lin L.G., Ye W.C. (2018). Techniques for Extraction and Isolation of Natural Products: A Comprehensive Review. Chin. Med..

[32] Wang L., Li Z., Huang J., Liu D., Lefebvre C., Fan J. (2022). Effect of Ultrasound-Assisted Extraction of Polyphenols from Apple Peels in Water CO_2_ Systems. Food Bioprocess Technol..

[33] Gonelimali F.D., Szabó-Nótin B., Máté M. (2021). Optimal Drying Conditions for Valorization of Industrial Apple Pomace: Potential Source of Food Bioactive Compounds. Prog. Agric. Eng. Sci..

[34] Bhat I.M., Wani S.M., Mir S.A., Naseem Z. (2023). Effect of Microwave-Assisted Vacuum and Hot Air Oven Drying Methods on Quality Characteristics of Apple Pomace Powder. Food Prod. Process. Nutr..

[35] Junjian R., Mingtao F., Yahui L., Guowei L., Zhengyang Z., Jun L. (2013). Optimisation of Ultrasonic-Assisted Extraction of Polyphenols from Apple Peel Employing Cellulase Enzymolysis. Int. J. Food Sci. Technol..

[36] Razola-Díaz M.d.C., Aznar-Ramos M.J., Guerra-Hernández E.J., García-Villanova B., Gómez-Caravaca A.M., Verardo V. (2022). Establishment of a Sonotrode Ultrasound-Assisted Extraction of Phenolic Compounds from Apple Pomace. Foods.

[37] Kawecka L., Galus S. (2021). Wytłoki Owocowe—Charakterystyka i Możliwości Zagospodarowanie. Postępy Tech. Przetwórstwa Spożywczego.

[38] Goli S.A.H., Ghaemifar M.M., Rezvani Z., Moradabbasi M. (2025). Valorization of Apple Pomace by Extraction of Insoluble Dietary Fiber and Production of IDF-Enriched Apple Juice Using Different Levels of Hydrocolloids. J. Food Meas. Charact..

[39] Thomas F. (2025). A Comparative Study of Three Drying Techniques for Apple Pomace Valorization Based on Key Performance Indicators. Master’s Thesis.

[40] Jha A.K., Sit N. (2022). Extraction of Bioactive Compounds from Plant Materials Using Combination of Various Novel Methods: A Review. Trends Food Sci. Technol..

[41] Zardo D.M., Alberti A., Zielinski A.A.F., Prestes A.A., Esmerino L.A., Nogueira A. (2021). Influence of Solvents in the Extraction of Phenolic Compounds with Antibacterial Activity from Apple Pomace. Sep. Sci. Technol..

[42] Lohani U.C., Muthukumarappan K. (2015). Effect of Drying Methods and Ultrasonication in Improving the Antioxidant Activity and Total Phenolic Content of Apple Pomace Powder. J. Food Res..

[43] Li W., Yang R., Ying D., Yu J., Sanguansri L., Augustin M.A. (2020). Analysis of Polyphenols in Apple Pomace: A Comparative Study of Different Extraction and Hydrolysis Procedures. Ind. Crops Prod..

[44] Zhang Z., Poojary M.M., Choudhary A., Rai D.K., Tiwari B.K. (2018). Comparison of Selected Clean and Green Extraction Technologies for Biomolecules from Apple Pomace. Electrophoresis.

[45] Bars-Cortina D., Macià A., Iglesias I., Garanto X., Badiella L., Motilva M.J. (2018). Seasonal Variability of the Phytochemical Composition of New Red-Fleshed Apple Varieties Compared with Traditional and New White-Fleshed Varieties. J. Agric. Food Chem..

[46] Elboughdiri N. (2018). Effect of Time, Solvent-Solid Ratio, Ethanol Concentration and Temperature on Extraction Yield of Phenolic Compounds from Olive Leaves. Eng. Technol. Appl. Sci. Res..

[47] Uysal S., Cvetanović A., Zengin G., Đurović S., Aktumsek A. (2017). Optimization of the Extraction Process of Antioxidants from Loquat Leaves Using Response Surface Methodology. J. Food Process. Preserv..

[48] Pollini L., Cossignani L., Juan C., Mañes J. (2021). Extraction of Phenolic Compounds from Fresh Apple Pomace by Different Non-Conventional Techniques. Molecules.

[49] Medina-Torres N., Ayora-Talavera T., Espinosa-Andrews H., Sánchez-Contreras A., Pacheco N. (2017). Ultrasound Assisted Extraction for the Recovery of Phenolic Compounds from Vegetable Sources. Agronomy.

[50] Kobus Z., Wilczyński K., Nadulski R., Rydzak L., Guz T. Effect of Solvent Polarity on the Efficiency of Ultrasound-Assisted Extraction of Polyphenols From Apple Pomace. Proceedings of the IX International Scientific Symposium “Farm Machinery and Processes Management in Sustainable Agriculture”.

[51] Krasnova I., Segliņa D. (2020). Content of Phenolic Compounds and Antioxidant Activity in Fresh Apple, Pomace and Pomace Water Extract—Effect of Cultivar. Proc. Latv. Acad. Sci. Sect. B Nat. Exact Appl. Sci..

[52] Egüés I., Hernandez-Ramos F., Rivilla I., Labidi J. (2021). Optimization of Ultrasound Assisted Extraction of Bioactive Compounds from Apple Pomace. Molecules.

[53] Chaovanalikit A., Mingmuang A., Kitbunluewit T., Choldumrongkool N., Sondee J., Chupratum S. (2012). Anthocyanin and Total Phenolics Content of Mangosteen and Effect of Processing on the Quality of Mangosteen Products. Int. Food Res. J..

[54] Tobiszewski M., Namieśnik J., Pena-Pereira F. (2017). Environmental Risk-Based Ranking of Solvents Using the Combination of a Multimedia Model and Multi-Criteria Decision Analysis. Green Chem..

[55] Silva E.M., Rogez H., Larondelle Y. (2007). Optimization of Extraction of Phenolics from Inga Edulis Leaves Using Response Surface Methodology. Sep. Purif. Technol..

[56] Yim H.S., Chye F.Y., Rao V., Low J.Y., Matanjun P., How S.E., Ho C.W. (2013). Optimization of Extraction Time and Temperature on Antioxidant Activity of Schizophyllum Commune Aqueous Extract Using Response Surface Methodology. J. Food Sci. Technol..

[57] Munteanu I.G., Apetrei C. (2021). Analytical Methods Used in Determining Antioxidant Activity: A Review. Int. J. Mol. Sci..

[58] Antony A., Farid M. (2022). Effect of Temperatures on Polyphenols during Extraction. Appl. Sci..

[59] Oancea S. (2021). A Review of the Current Knowledge of Thermal Stability of Anthocyanins and Approaches to Their Stabilization to Heat. Antioxidants.

[60] Ioannou I., Chekir L., Ghoul M. (2020). Effect of Heat Treatment and Light Exposure on the Antioxidant Activity of Flavonoids. Processes.

[61] Frontuto D., Carullo D., Harrison S.M., Brunton N.P., Ferrari G., Lyng J.G., Pataro G. (2019). Optimization of Pulsed Electric Fields-Assisted Extraction of Polyphenols from Potato Peels Using Response Surface Methodology. Food Bioprocess Technol..

[62] Maghsoudlou Y., Asghari Ghajari M., Tavasoli S. (2019). Effects of Heat Treatment on the Phenolic Compounds and Antioxidant Capacity of Quince Fruit and Its Tisane’s Sensory Properties. J. Food Sci. Technol..

[63] Larrauri J.A., Rupérez P., Saura-Calixto F. (1997). Effect of Drying Temperature on the Stability of Polyphenols and Antioxidant Activity of Red Grape Pomace Peels. J. Agric. Food Chem..

[64] Táborský J., Sus J., Lachman J., Šebková B., Adamcová A., Šatínský D. (2021). Dynamics of Phloridzin and Related Compounds in Four Cultivars of Apple Trees during the Vegetation Period. Molecules.

[65] Hrubá M., Baxant J., Čížková H., Smutná V., Kovařík F., Ševčík R., Hanušová K., Rajchl A. (2021). Phloridzin as a Marker for Evaluation of Fruit Product’s Authenticity. Czech J. Food Sci..

[66] Ni T., Zhang S., Rao J., Zhao J., Huang H., Liu Y., Ding Y., Liu Y., Ma Y., Zhang S. (2024). Phlorizin, an Important Glucoside: Research Progress on Its Biological Activity and Mechanism. Molecules.

[67] Orozco-Flores L.A., Salas E., Rocha-Gutiérrez B., Peralta-Pérez M.D.R., González-Sánchez G., Ballinas-Casarrubias L. (2024). Determination of Polyphenolic Profile of Apple Pomace (Malus Domestica Golden Delicious Variety) by HPLC-MS. ACS Omega.

[68] Bai X.L., Yue T.L., Yuan Y.H., Zhang H.W. (2010). Optimization of Microwave-Assisted Extraction of Polyphenols from Apple Pomace Using Response Surface Methodology and HPLC Analysis. J. Sep. Sci..

[69] Markowski J., Płocharski W. (2006). Determination of Phenolic Compounds in Apples and Processed Apple Products. J. Fruit Ornam. Plant Res..

[70] Skrypnik L., Novikova A. (2020). Response Surface Modeling and Optimization of Polyphenols Extraction from Apple Pomace Based on Nonionic Emulsifiers. Agronomy.

[71] Klimczak I., Małecka M., Szlachta M., Gliszczyńska-Świgło A. (2007). Effect of Storage on the Content of Polyphenols, Vitamin C and the Antioxidant Activity of Orange Juices. J. Food Compos. Anal..

[72] Tabaszewska M., Najgebauer-Lejko D. (2020). The Content of Selected Phytochemicals and in Vitro Antioxidant Properties of Rose Hip (*Rosa canina* L.) Tinctures. NFS J..

